# Supplementary material for: Comparative genomics provides new insights into the diversity, physiology, and sexuality of the only industrially exploited tremellomycete: Phaffia rhodozyma
Source: BMC Genomics. 2016 Nov 9;17:901. doi: 10.1186/s12864-016-3244-7 (PMC5103461; doi:10.1186/s12864-016-3244-7)
Supplement: Additional file 6: — List of orphan genes with links to PFAM (related to Additional file 1: Table S1). (ZIP 1428 kb) [file 12864_2016_3244_MOESM6_ESM.zip › BLAST_HTML_FTR/G05467_P.html]

BLAST Search Results


```
BLASTP 2.2.27+


Reference:
Stephen F. Altschul, Thomas L. Madden, Alejandro A. Schäffer,
Jinghui Zhang, Zheng Zhang, Webb Miller, and David J. Lipman (1997),
"Gapped BLAST and PSI-BLAST: a new generation of protein database
search programs", Nucleic Acids Res. 25:3389-3402.


Reference for
composition-based statistics:
Alejandro A. Schäffer, L. Aravind, Thomas L. Madden, Sergei
Shavirin, John L. Spouge, Yuri I. Wolf, Eugene V. Koonin, and
Stephen F. Altschul (2001), "Improving the accuracy of PSI-BLAST
protein database searches with composition-based statistics and
other refinements", Nucleic Acids Res. 29:2994-3005.


Database: nr
           71,551,133 sequences; 26,053,659,533 total letters


Query= G05467_P

Length=127
                                                                      Score     E
Sequences producing significant alignments:                          (Bits)  Value

emb|CDZ98881.1|  hypothetical protein [Xanthophyllomyces dendrorh...   213    6e-68
ref|WP_051768367.1|  hypothetical protein [Streptomyces sp. NRRL ...  36.2    1.4  
ref|WP_037916592.1|  tetratricopeptide repeat protein [Streptomyc...  36.6    1.8  
gb|ESZ88098.1|  sulfatase [Blastomonas sp. CACIA14H2]                 37.4    2.0  
ref|WP_039428661.1|  tetratricopeptide repeat protein [Porphyromo...  35.4    8.3  
ref|WP_019098858.1|  MULTISPECIES: hypothetical protein [Pseudomo...  35.8    8.3  
ref|WP_016713310.1|  hypothetical protein [Pseudomonas monteilii]     35.8    8.3  
ref|WP_053691599.1|  hypothetical protein [Streptomyces sp. WM637...  34.7    8.3  


 >emb|CDZ98881.1| hypothetical protein [Xanthophyllomyces dendrorhous]
Length=138

 Score =  213 bits (542),  Expect = 6e-68, Method: Compositional matrix adjust.
 Identities = 110/117 (94%), Positives = 111/117 (95%), Gaps = 1/117 (1%)

Query  1    MASSTISPFLSLPVVLSLLSAEPESIPLLSALAFFLSQDPSPDSLRLPGTPSLHQVRLRL  60
            MASSTISPFLSLPVVLSLLSAEPESIPLLSALAFFLSQDPSPDSLRLPGTPSLHQVRLRL
Sbjct  1    MASSTISPFLSLPVVLSLLSAEPESIPLLSALAFFLSQDPSPDSLRLPGTPSLHQVRLRL  60

Query  61   AQTFINHDRLSEAESELKLDERETRKARTEEWSECVGMLGDVLERMGRTGQARTWRA  117
            AQTFINHDRLSEAESELKLDERETRKARTEEWSECVGMLGDVLERMG+    RT  A
Sbjct  61   AQTFINHDRLSEAESELKLDERETRKARTEEWSECVGMLGDVLERMGQ-ANVRTLVA  116


>ref|WP_051768367.1| hypothetical protein [Streptomyces sp. NRRL F-6131]
Length=97

 Score = 36.2 bits (82),  Expect = 1.4, Method: Compositional matrix adjust.
 Identities = 29/85 (34%), Positives = 43/85 (51%), Gaps = 10/85 (12%)

Query  34   FFLSQDPSPDSLR-----LPGTPSLHQVRLRLAQTFINHDRLSEAESELKLDERETRKAR  88
            F+       D+ R     L   P+   VRL LA+++ +  RL +AE+EL+   R      
Sbjct  4    FYFDSKAYTDAARMLEGVLAEEPANLSVRLLLARSYFHSARLGKAEAELR---RVLDADP  60

Query  89   TEEWSECVGMLGDVLERMGRTGQAR  113
            TE+++    MLG  LER GR  +AR
Sbjct  61   TEDYARL--MLGRTLERQGRHEEAR  83


>ref|WP_037916592.1| tetratricopeptide repeat protein [Streptomyces sp. PCS3-D2]
 gb|EYU70859.1| hypothetical protein AW27_04020 [Streptomyces sp. PCS3-D2]
Length=116

 Score = 36.6 bits (83),  Expect = 1.8, Method: Compositional matrix adjust.
 Identities = 26/60 (43%), Positives = 32/60 (53%), Gaps = 11/60 (18%)

Query  57   RLRLAQTFINHDRLSEAESELKLDERETRKARTEEW---SECVGMLGDVLERMGRTGQAR  113
            RL LA+ F +  +LS AE EL+        A  E W        MLG  LER+GRTG+AR
Sbjct  50   RLLLARAFYHSAQLSRAERELR--------AVLERWPVEDYARLMLGRTLERLGRTGEAR  101


>gb|ESZ88098.1| sulfatase [Blastomonas sp. CACIA14H2]
Length=474

 Score = 37.4 bits (85),  Expect = 2.0, Method: Composition-based stats.
 Identities = 30/121 (25%), Positives = 53/121 (44%), Gaps = 5/121 (4%)

Query  5    TISPFLSLPVVLSLLSAEPESIPLLSALA----FFLSQDPSPDSLRLPGTPSLHQVRLRL  60
            + SP  S+ +  ++L A     P+ + L        +Q P PD L + G   ++  RL  
Sbjct  342  SASPVSSIDIAATVLRAADPGQPVPTELEGHDLVATAQRPQPDRLLIWGQEPVYAARLGS  401

Query  61   AQTFINHDRLSEAESELKLDERETRKARTEEWSECVGMLGDVLERMGRTGQARTWRAKLS  120
             + +++HD       +LK D  ET+     +       LGD LE+  ++  A  W   L+
Sbjct  402  KKFWVSHDWKQSYLYDLKGDPTETKDLSAAD-PATAKQLGDALEKFRKSLPAPLWELHLT  460

Query  121  K  121
            +
Sbjct  461  R  461


>ref|WP_039428661.1| tetratricopeptide repeat protein [Porphyromonas sp. COT-052 OH4946]
 gb|KGL56227.1| tetratricopeptide repeat protein [Porphyromonas sp. COT-052 OH4946]
Length=337

 Score = 35.4 bits (80),  Expect = 8.3, Method: Compositional matrix adjust.
 Identities = 35/112 (31%), Positives = 52/112 (46%), Gaps = 15/112 (13%)

Query  15   VLSLLSAEPESIPLLSALAFFLSQDPSPDSLR-----LPGTPSLHQVRLRLAQTFINHDR  69
            +++ L AE E +P+L A  F L   P  ++++     L   P   +    +A   I   R
Sbjct  213  LVAALDAEEEQVPMLRARIFMLEGKPQ-NAMQAYEELLSLDPFNEEGHRGIASLMIKDGR  271

Query  70   LSEAESELKLDERETRKARTE--EWSECVGMLGDVLERMGRTGQARTWRAKL  119
             +EAE  L       R+A  E  E      ML ++LE  G+T +A  WRAKL
Sbjct  272  SAEAEKFL-------REAIEEMPEQRNLTLMLVEILENQGQTKEADEWRAKL  316


>ref|WP_019098858.1| MULTISPECIES: hypothetical protein [Pseudomonas]
Length=573

 Score = 35.8 bits (81),  Expect = 8.3, Method: Compositional matrix adjust.
 Identities = 35/116 (30%), Positives = 55/116 (47%), Gaps = 28/116 (24%)

Query  3    SSTISPFLSLPVVLSLLSAEPESIPLL-SALAFFLSQDPSPDSLRLPGTPSLHQVRLRLA  61
            +  I+P L    +L  L   PE++PLL SA+        +PD  RL         RL  A
Sbjct  215  NGEIAPILLRARLLQTLDRGPEALPLLRSAIR------DNPDDKRL---------RLTYA  259

Query  62   QTFINHDRLSEAESE-LKL-----DERETRKA------RTEEWSECVGMLGDVLER  105
            +T +  DR+++A+ E L L     D+ E R +        ++W E  G L +++ER
Sbjct  260  RTLVEQDRIADAKGEFLSLVQQYPDDDELRYSLALVCLENKDWDEAEGYLQELIER  315


>ref|WP_016713310.1| hypothetical protein [Pseudomonas monteilii]
Length=573

 Score = 35.8 bits (81),  Expect = 8.3, Method: Compositional matrix adjust.
 Identities = 35/116 (30%), Positives = 55/116 (47%), Gaps = 28/116 (24%)

Query  3    SSTISPFLSLPVVLSLLSAEPESIPLL-SALAFFLSQDPSPDSLRLPGTPSLHQVRLRLA  61
            +  I+P L    +L  L   PE++PLL SA+        +PD  RL         RL  A
Sbjct  215  NGEIAPILLRARLLQTLDRGPEALPLLRSAIR------DNPDDKRL---------RLTYA  259

Query  62   QTFINHDRLSEAESE-LKL-----DERETRKA------RTEEWSECVGMLGDVLER  105
            +T +  DR+++A+ E L L     D+ E R +        ++W E  G L +++ER
Sbjct  260  RTLVEQDRIADAKGEFLSLVQQYPDDDELRYSLALVCLENKDWDEAEGYLQELIER  315


>ref|WP_053691599.1| hypothetical protein [Streptomyces sp. WM6372]
 gb|KOU22089.1| tetratricopeptide repeat protein [Streptomyces sp. WM6372]
Length=116

 Score = 34.7 bits (78),  Expect = 8.3, Method: Compositional matrix adjust.
 Identities = 25/58 (43%), Positives = 35/58 (60%), Gaps = 7/58 (12%)

Query  57   RLRLAQTFINHDRLSEAESELK-LDERETRKARTEEWSECVGMLGDVLERMGRTGQAR  113
            RL LA+ + +  RLS AE EL+ + ER       E++++   MLG  LER GR G+AR
Sbjct  50   RLLLARAYYHSARLSRAEHELRAILERWP----VEDYAQL--MLGRTLERQGRAGEAR  101


Lambda      K        H        a         alpha
   0.319    0.133    0.387    0.792     4.96 

Gapped
Lambda      K        H        a         alpha    sigma
   0.267   0.0410    0.140     1.90     42.6     43.6 

Effective search space used: 637819150023


  Database: nr
    Posted date:  Sep 23, 2015 12:05 AM
  Number of letters in database: 26,053,659,533
  Number of sequences in database:  71,551,133


Matrix: BLOSUM62
Gap Penalties: Existence: 11, Extension: 1
Neighboring words threshold: 11
Window for multiple hits: 40
```
